# Noninvasive Diagnosis of the Mitochondrial Function of Doxorubicin-Induced Cardiomyopathy Using In Vivo Dynamic Nuclear Polarization–Magnetic Resonance Imaging

**DOI:** 10.3390/antiox11081454

**Published:** 2022-07-26

**Authors:** Yukie Mizuta, Tomohiko Akahoshi, Hinako Eto, Fuminori Hyodo, Masaharu Murata, Kentaro Tokuda, Masatoshi Eto, Ken Yamaura

**Affiliations:** 1 Operating Rooms, Kyushu University Hospital, Fukuoka 812-8582, Japan; 2 Department of Anesthesiology and Critical Care Medicine, Graduate School of Medical Sciences, Kyushu University, Fukuoka 812-8582, Japan; yamaura.ken.361@m.kyushu-u.ac.jp; 3 Department of Disaster and Emergency Medicine, Graduate School of Medical Sciences, Kyushu University, Fukuoka 812-8582, Japan; akahoshi.tomohiko.006@m.kyushu-u.ac.jp; 4 Center for Advanced Medical Innovation, Kyushu University, Fukuoka 812-8582, Japan; etohinako@camiku.kyushu-u.ac.jp (H.E.); m-murata@camiku.kyushu-u.ac.jp (M.M.); etom@uro.med.kyushu-u.ac.jp (M.E.); 5 Department of Radiology, Frontier Science for Imaging, School of Medicine, Gifu University, Gifu 501-1194, Japan; hyodof@gifu-u.ac.jp; 6 Department of Anesthesiology and Critical Care Medicine, Kyushu University Beppu Hospital, Beppu 874-0838, Japan; tokuda.kentaro.160@m.kyushu-u.ac.jp

**Keywords:** dynamic nuclear polarization–magnetic resonance imaging, oxidation reduction, mitochondria, nitroxyl radical, heart

## Abstract

Doxorubicin (DOX) induces dose-dependent cardiotoxicity via oxidative stress and abnormal mitochondrial function in the myocardium. Therefore, a noninvasive in vivo imaging procedure for monitoring the redox status of the heart may aid in monitoring diseases and developing treatments. However, an appropriate technique has yet to be developed. In this study, we demonstrate a technique for detecting and visualizing the redox status of the heart using in vivo dynamic nuclear polarization–magnetic resonance imaging (DNP–MRI) with 3-carbamoyl-PROXYL (CmP) as a molecular imaging probe. Male C57BL/6N mice were administered DOX (20 mg/kg) or saline. DNP–MRI clearly showed a slower DNP signal reduction in the DOX group than in the control group. Importantly, the difference in the DNP signal reduction rate between the two groups occurred earlier than that detected by physiological examination or clinical symptoms. In an in vitro experiment, KCN (an inhibitor of complex IV in the mitochondrial electron transport chain) and DOX inhibited the electron paramagnetic resonance change in H9c2 cardiomyocytes, suggesting that the redox metabolism of CmP in the myocardium is mitochondrion-dependent. Therefore, this molecular imaging technique has the potential to monitor the dynamics of redox metabolic changes in DOX-induced cardiomyopathy and facilitate an early diagnosis of this condition.

## 1. Introduction

Doxorubicin (DOX; Adriamycin) is an anthracycline that is extensively used in clinical practice as an effective anticancer agent [1]. A major side effect of this class of chemotherapeutic drugs is dose-dependent cardiotoxicity, which leads to dilated cardiomyopathy and heart failure [2,3]. Once DOX-induced cardiomyopathy develops, it has a poor prognosis and is often fatal [4]. Moreover, in the early stages of DOX-induced cardiomyopathy, it is difficult to predict whether a patient will develop heart failure [5]. Other than endomyocardial biopsy, there are no methods to detect cardiomyopathy in its early stages [6]. However, biopsy is an invasive procedure that invites the risk of ventricular perforation [7]. Therefore, a diagnostic in vivo imaging technique must be developed.

As is well known, cardiomyocytes are rich in mitochondria. Naturally, oxidative stress and abnormal mitochondrial function are involved in the pathogenesis of DOX-induced cardiomyopathy [8,9,10]. Therefore, an in vivo imaging technique that facilitates noninvasive detection of mitochondrial oxidative stress and abnormalities may be useful for diagnosing cardiomyopathy in its early stages. 

In vivo dynamic nuclear polarization–magnetic resonance imaging (DNP–MRI) is a method that allows free-radical imaging in living animals [11,12]. The enhancement in the MR image intensity derived from free radicals, which is an effect of DNP, can be obtained by electron paramagnetic resonance (EPR) irradiation of free radical molecules prior to the MRI pulse sequence [11,13,14]. Stable, low-molecular weight nitroxyl radicals, including 3-carbamoyl-2,2,5,5-tetramethyl-1-pyrrolidine-1-oxyl, which is also known as 3-carbamoyl-PROXYL (CmP), are less toxic [15], sensitively react with redox molecules within a tissue, and have been used as probes in various biophysical and biochemical experiments [16,17,18,19,20,21,22,23,24]. After the nitroxyl radical undergoes redox reaction in tissues, it loses its free radical and is mainly converted to its reduced form, hydroxylamine, resulting in the decreased enhancement of image intensity in DNP–MRI [25]. A recent study suggested that the redox reaction of CmP is related to mitochondrial electron transport chain (ETC) dysfunction and reduced antioxidant species [26,27]. Our previous studies using in vivo DNP–MRI with nitroxyl radicals demonstrated a relationship between redox balance and disorders such as liver fibrosis, hepatitis, and acute muscle damage [24,25,26,27].

Instrumentation and techniques for EPR imaging of the heart are currently under development [28,29,30,31]. Detection of free radical changes in cases of cardiac disease using nitroxyl radicals has only been attempted in ex vivo studies [32,33,34]; the detection of these changes is not reported in in vivo studies using pathological models. In addition, whether DNP–MRI can be used to determine the relationship between redox balance and cardiomyopathy and for early diagnosis of heart failure remains unclear. In this study, we evaluated the feasibility of assessing the in vivo redox status assessed using DNP–MRI with a CmP probe for monitoring DOX-induced cardiomyopathy.

## 2. Materials and Methods 

### 2.1. Animal Experiments

Eight- to nine-week-old male C57BL/6N mice weighing 20–25 g were purchased from Charles River Laboratories Japan, Inc. (Yokohama, Japan). The mice were maintained under controlled conditions (22 ± 2 °C and 12-h light/dark cycle) in chambers and provided with water and food ad libitum. All animal experiments were approved by the Ethics Committee for Animal Experiments of Kyushu University (approval number: A21-180-0). All methods were performed in accordance with the guidelines for animal experiments drafted by Kyushu University. Moreover, this manuscript complies with the Uniform Requirements for manuscripts submitted to biomedical journals, published by the International Committee of Medical Journal Editors. The sample size (number of mice needed) was calculated as the minimum number required to reach significance according to the 3R “Replace, Reduce, Refine” rules. Sample size was calculated by comparing the control and DOX groups using the *t*-test for two independent means (two groups) to evaluate the variance between the groups. A *p*-value < 0.05 was considered statistically significant.

### 2.2. DOX-Induced Cardiomyopathy Mouse Model

The mice (n = 10) were intraperitoneally injected with 20 mg/kg DOX (doxorubicin; Wako, Tokyo, Japan). The control group (n = 10) was intraperitoneally injected with the same volume of saline solution. Mice were monitored for survival for up to 6 days after DOX injection. They were considered lethally induced when they could no longer consume food or water or if they lost >20% of their initial body weight, at which time they were euthanized with 5% isoflurane and cervical dislocation. These cases were recorded as DOX-induced mortality. Cardiac function was measured via echocardiography before injecting DOX and 2 and 6 days later in the survived mice. The mice were euthanized after echocardiography on day 6, and the heart tissue was collected for analysis.

### 2.3. Echocardiographic Measurements

A 13-MHz linear array ultrasound probe (Hitachi EUB5500 and EUP-L54MA; Hitachi, Tokyo, Japan) was used to perform transthoracic echocardiography. The mice were anesthetized with 1–2% isoflurane, and their heart rates were maintained between 400 and 450 beats per minute [35]. The left ventricular systolic dimensions (LVDd) and left ventricular diastolic dimensions (LVDs) were measured in the short-axis view using conventional M-mode echocardiography. Left ventricular end-diastolic volume (LVEDV) and left ventricular end-systolic volume (LVESV) were calculated using the Teichholz formula: volume = 7.0 × dimension^3^/(2.4 + dimension). The percentages of LV ejection fraction (EF) and LV fractional shortening (FS) were calculated using the following formula: LVEF = (LVEDV − LVESV)/LVEDV × 100 and LVFS = (LVDd − LVDs)/LVDd × 100. All measurements were averaged over three consecutive cardiac cycles by an experienced technician who was blinded to the study groups.

### 2.4. Histopathology

Heart tissues were fixed with 10% formalin, paraffin-embedded, and cut into 6-μm sections. For histological assessment, the sections were stained with hematoxylin and eosin (H&E). These sections were routinely deparaffinized and evaluated for focal cytoplasmic vacuolization, which can be observed in DOX-induced cardiac injury and indicates cell death [36,37]. The heart sections were also routinely deparaffinized and immunostained for 8-hydroxy-2′-deoxyguanosine (8-OHdG), and then microwaved in 10 mM citrate buffer (pH 6.0) for 15 min to retrieve the antigens. After washing the sections three times with phosphate-buffered saline (PBS), they were sequentially treated with 3% skimmed milk in PBS for 30 min at room temperature to block non-specific binding, 1 μg/mL of anti-8-OHdG polyclonal antibody (Bioss Antibodies, Woburn, MA, USA), and a peroxidase-conjugated avidin complex as the secondary antibody (EnVision + kits; Dako Japan Co. Ltd., Kyoto, Japan). The stained sections were visualized using Axio Scan Z1 and Zen (Carl Zeiss AG. Ltd., Thornwood, NY, USA). The brown color relative to the entire area was measured as the 8-OHdG-positive area in five random fields using ImageJ software version 1.50i (National Institutes of Health, Bethesda, MD, USA).

### 2.5. In Vivo Redox Imaging of the Heart Using DNP–MRI

In vivo redox imaging was performed using an in vivo DNP–MRI system (Keller imaging system; Japan REDOX Inc., Fukuoka, Japan). The external magnetic field B_0_ for EPR irradiation and MRI was fixed at 16 mT, and the frequencies of the EPR irradiation and MRI were 455 MHz and 683 kHz, respectively. For EPR irradiation during cardiac imaging, a one-turn, curved-surface, rectangular coil (longitudinal, 20 mm; lateral, 32 mm) was constructed. The scanning conditions for in vivo DNP–MRI were as follows: power of EPR irradiation, 11 W; flip angle, 90°; repetition time × echo time × EPR irradiation time, 500 × 25 × 250 ms; number of averages, 2; slice thickness, 50 mm including the entire width of each mouse; phase-encoding steps, 32; field of view, 40 × 40 nm; and matrix size, 64 × 64 after reconstruction. In the in vivo experiments, 2 and 6 days after the administration of DOX or saline, mice were anesthetized with 2% isoflurane and secured in sternal recumbency on a specialized holder with adhesive skin tape. During the procedure, the body temperature of mice was maintained at 37 ± 1 °C using a heating pad. The holder was placed in the resonator, and in vivo DNP–MRI of the thorax was started immediately after intravenous administration of CmP (150 mM, 10 μL/g; Aldrich Chemical Co., Milwaukee, WI, USA). Pharmacokinetic DNP–MRI images were obtained at several time points from 1 to 13 min post-injection. Normal MRI images were obtained without EPR irradiation. The image enhancement decay rate of each mouse was obtained from the slope of the average image intensity in the region of interest (ROI), equivalent to the enhancement by CmP, using ImageJ software. 

### 2.6. Quantitative Analysis of Oxidized and Total CmP Concentration in the Heart and Blood

CmP solution (150 mM, 10 μL/g) was intravenously injected into the mice 2 days after injecting DOX or saline. Mice were sacrificed to collect the heart tissue and blood 5 min after injecting CmP. Fresh heart tissue was homogenized in half saline solution using a Potter—Elvehjem grinder on ice. The total CmP was reoxidized by adding potassium ferricyanide (2 mM) to the heart tissue and blood, and it was measured using an X-band EPR spectrometer (JEOL, Tokyo, Japan). The measurement parameters for the X-band EPR were as follows: microwave frequency, 1.0 GHz; microwave power, 1.0 mW; center of field, 335 mT; modulation width, 0.04 mT; sweep time, 1 min; sweep width, 5.0 mT; and time constant, 0.03 s.

### 2.7. H9c2 Cell Culture

The rat myocardial cell line H9c2 (CRL-1446) was purchased from American Type Culture Collection (Manassas, VA, USA). Cells were cultured in Dulbecco’s modified Eagle’s medium (DMEM, referred to as high-glucose media; Wako, Tokyo, Japan) supplemented with Antibiotic–Antimycotic (Gibco, Thermo Fisher Scientific, Waltham, MA, USA) and 10% fetal bovine serum (FBS, Gibco). The cell culture was maintained at 37 °C in a humidified atmosphere containing 5% CO_2_. H9c2 cells were passaged when they reached approximately 80% confluence, and the medium was changed every 2–3 days. Cells from passages six to eight were used in this study.

### 2.8. Cell Treatments and Assessment of CmP Dynamics

H9c2 cells were seeded at a density of 2 × 10^6^ cells/flask in a 75-cm^2^ flask with 10% FBS and incubated for 24 h. Subsequently, the medium was replaced with DMEM supplemented with 1% FBS [38] to arrest cell proliferation prior to the experiments to be performed, in accordance with a previous study [39]. After 24 h, the cells were treated with DOX (0.1 μM; Wako) for 24 h prior to analysis. Control cells were treated with the same volume of saline solution in the media. The cells were then rinsed with PBS, dissociated from the dish using 4 mL of Accutase (Innovative Cell Technologies, San Diego, CA, USA), and homogenized with half saline on ice. The reduction rate of CmP radicals was measured using X-band EPR 5 min after mixing CmP (20 μM) and H9c2 homogenates (×4). Upon the addition of potassium cyanide (KCN; Wako), an inhibitor of complex IV in the mitochondrial ETC, CmP radicals were also monitored. The reduction rate was calculated from the peak height of the CmP radical spectra after 30 min. The measurement parameters for X-band EPR were as follows: microwave frequency, 1.0 GHz; microwave power, 1.0 mW; center of field, 330 mT; modulation width, 0.04 mT; sweep time, 1 min; sweep width, 5.0 mT; and time constant, 0.03 s.

### 2.9. Oxygen Consumption Rate Assays

A Mitochondrial Stress Test Complete Assay Kit (Abcam, Cambridge, UK; cat. no. ab232857) was used to assess oxygen consumption rate (OCR), which is considered a parameter of mitochondrial function, according to the manufacturer’s protocol. Briefly, H9c2 cells were seeded at a density of 80,000 cells/well in 96-well plates and incubated overnight. The cells were treated with DOX (0.1 μM) or the same volume of saline for 24 h prior to analysis. The medium was then replaced, and an extracellular O_2_ probe (10 μM) was added to each well. The oxygen consumption rate was calculated by determining the increase in fluorescent signals (excitation wavelength of 380 nm and emission wavelength of 650 µm), which were measured using an Infinite M1000 microplate reader (Tecan, Zurich, Switzerland) and i-control software (Tecan).

### 2.10. Statistical Analysis

The data are presented as the mean ± standard error of the mean. Differences among the four groups were evaluated using one-way analysis of variance (ANOVA) followed by Tukey’s multiple comparison test, and echocardiographic data were analyzed using two-way ANOVA followed by Tukey’s post hoc test using software Prism 8 (GraphPad Software, La Jolla, CA, USA). A Kaplan–Meier survival analysis was performed using the log-rank test. A *p*-value < 0.05 was considered statistically significant.

## 3. Results

**Figure 1:**
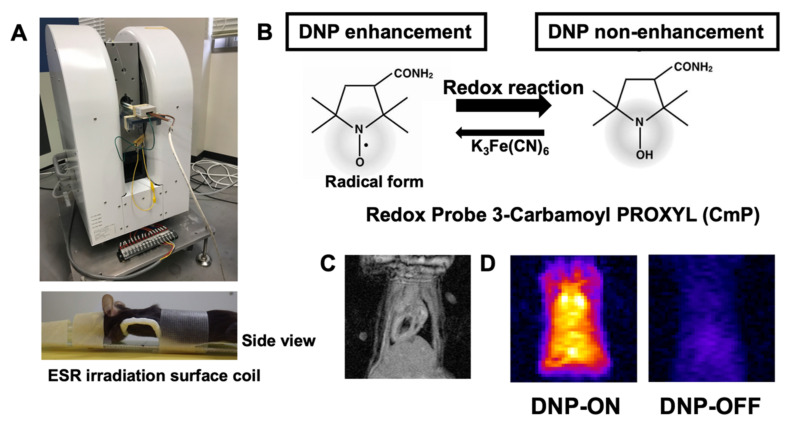
Redox imaging of the mouse heart after injection of nitroxyl radical. (**A**) Experimental set-up using the in vivo DNP–MRI system (Keller). (**B**) Chemical structure of the redox probe, 3-carbamoyl-PROXYL, and its nitroxyl reduction in the tissue, indicating the redox status. (**C**) Cardiac imaging by 1.5 T animal MRI. (**D**) In vivo DNP–MRI images of the heart 1 min after injecting 3-carbamoyl-PROXYL with DNP ON and OFF (i.e., with and without EPR irradiation, respectively).

In vivo redox imaging of the heart was performed using an in vivo DNP–MRI system constructed in our laboratory (Figure 1A). For EPR irradiation, a one-turn, curved-surface, rectangular coil was used as a highly sensitive detector to image the heart in this study (Figure 1A). To visualize the redox status of the heart, CmP was used as a source of nitroxyl radicals, which enable intracellular redox metabolism (Figure 1B). We confirmed that the signal intensity was clearly enhanced by EPR irradiation (DNP ON) in the hearts of the mice, although it was not enhanced without EPR irradiation (DNP OFF) (Figure 1C,D).

**Figure 2:**
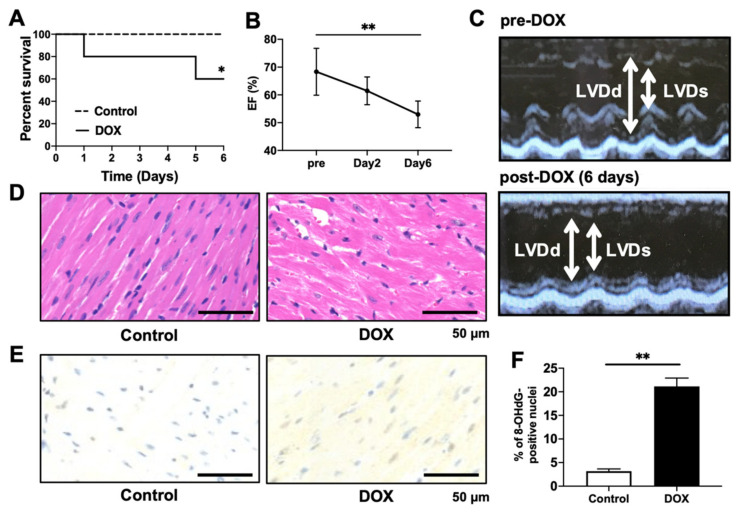
Effects of DOX injection on survival, cardiac function, and histology. (**A**) Kaplan–Meier survival curve showing survival after DOX injection. n = 10. * *p =* 0.0392 vs. control group. (**B**,**C**) Echocardiography performed in mice before DOX injection at 2 and 6 days after DOX injection. (C) Representative images of 2D M-mode echocardiograms. Ejection fractions (EFs) were quantified. n = 6 in each group. ** *p* < 0.01. (**D**) Representative photomicrographs of heart sections with H&E staining 6 days after DOX injection. Scale bar, 50 μm. (**E**,**F**) Immunohistochemical detection of oxidative DNA damage with 8-OHdG. Representative photomicrographs of the 8-OHdG-positive nuclei. Scale bar, 50 μm. 8-OHdG-positive nuclei/total cells (%) were normalized to the control group. n = 5 in each group. ** *p* < 0.01.

To confirm the acute phase toxicity of DOX in mice, we monitored their survival for up to 6 days. Mice that were intraperitoneally injected with DOX (20 mg/kg) showed signs of heart failure, including hypotension, bradycardia, and weight loss (data not shown). Of the total mice, 40% were euthanized (Figure 2A). To measure the changes in cardiac function after DOX administration, echocardiography was performed in mice before DOX injection and 2 and 6 days later. DOX administration gradually decreased the LVEF with the passage of time (Figure 2B,C). To examine DOX-induced cardiomyocyte injury, we analyzed heart sections via H&E staining. The heart tissues of DOX-treated mice exhibited myocardial swelling, myofibrillar loss, perinuclear vacuolization, and cytoplasmic vacuolization (Figure 2D). Immunohistochemistry was also performed to detect 8-OHdG, a critical marker of oxidative DNA damage. DOX increased the percentage of 8-OHdG-positive nuclei (Figure 2E,F).

**Figure 3:**
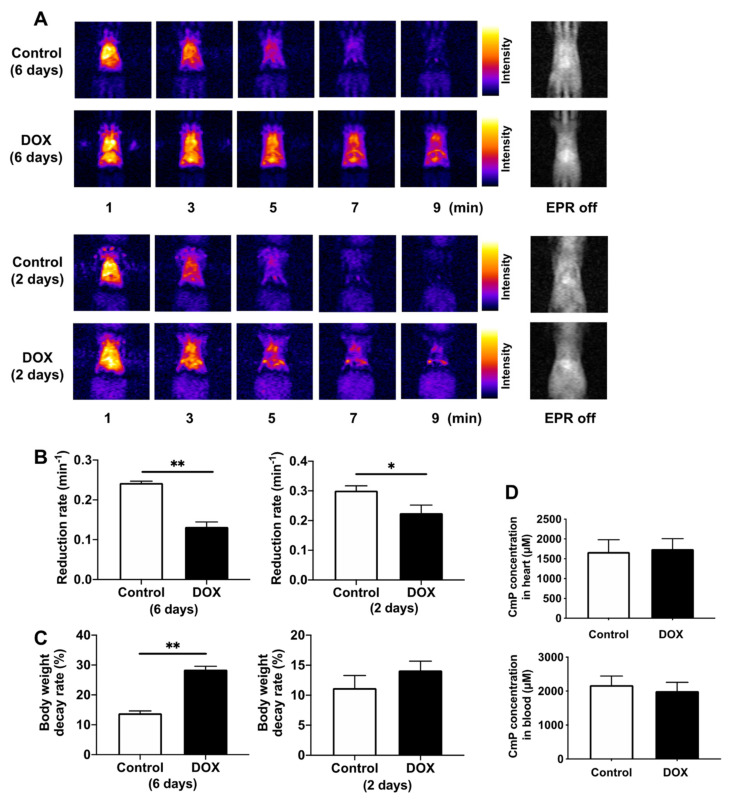
Redox imaging of the hearts of DOX-treated mice using in vivo DNP–MRI. (**A**) Representative imaging of temporal changes in DNP–MRI after intravenous injection of CmP at days 2 and 6 after DOX injection. (**B**) Comparison of the reduction rate of DNP image intensity in control and DOX-treated mice. Reduction rates were calculated by the slope of image intensity in the region of interest corresponding to the enhancement by CmP. n = 5–6 in each group. * *p* < 0.05, ** *p* < 0.01. (**C**) Comparison of body weight decay rate in control and DOX-treated mice at days 2 and 6 post-DOX injection. n = 5–6 in each group. ** *p* < 0.01. (**D**) Analysis of the total CmP radical concentration (sum of oxidized and reduced forms) in the heart and blood. Total CmP concentration was obtained after reoxidation by treating the heart tissue and blood samples with 2 mM potassium ferricyanide via X-band EPR. n = 4 in each group.

Next, we assessed the in vivo redox status of the hearts of DOX-treated mice using in vivo DNP–MRI. We confirmed that the distribution of injected CmP was similar in both the control and DOX-treated mice. CmP-induced enhancement of image intensity gradually decreased over time (Figure 3A). The reduction rate of image intensity in DOX-treated mice on day 6 after administration was significantly lower than that of control mice, and the body weight decay rate was also significantly higher (Figure 3B,C). In contrast, the reduction rate of image intensity in DOX-treated mice on day 2 after administration was significantly lower than that of control mice, although the body weight decay rate did not change significantly (Figure 3B,C). Considering the possibility that changes in the cardiac output and drug metabolism may alter the distribution of the radical probe, the oxidized form and total CmP concentration (sum of reduced and oxidized forms) in the heart tissue and blood were measured quantitatively using X-band EPR to confirm that they did not change in either group (Figure 3D). 

**Figure 4:**
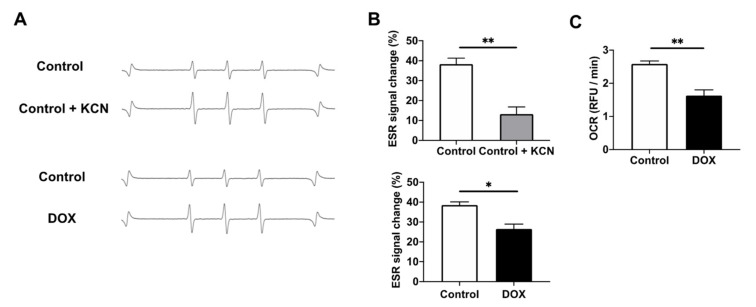
Assessment of CmP dynamics in H9c2 cells. (**A**) A typical ESR signal attenuation of CmP in four-fold diluted H9c2 cells homogenate solution over 30 min. (**B**) Comparison of the reduction rate of CmP radical concentration over 30 min in control H9c2 cells vs. KCN-treated H9c2 cells and control H9c2 cells vs. DOX-treated H9c2 cells. CmP concentration was measured by X-band EPR. n = 3 in each group. * *p* < 0.05, ** *p* < 0.01. (**C**) Oxygen consumption rate (OCR) over 82 min calculated using relative fluorescence unit slopes in control H9c2 cells vs. DOX-treated H9c2 cells. n = 3 in each group. ** *p* < 0.01.

To clarify the mechanisms of the redox reaction between CmP and the myocardium, H9c2 cell homogenates were directly monitored by X-band EPR. The EPR signal of CmP was reduced in H9c2 homogenates. The EPR signal change at 30 min was significantly inhibited by adding KCN, an inhibitor of complex IV in the mitochondrial ETC (Figure 4A,B). These data suggest that CmP redox metabolism in H9c2 cells is mitochondrion dependent. The EPR signal change at 30 min was also significantly lower in DOX-treated H9c2 cells than in the control (Figure 4A,B). Taken together, these results suggest that the CmP redox metabolism of DOX-treated H9c2 cells was decreased because of mitochondrial dysfunction. To elucidate the mitochondrial function, we measured the OCR of H9c2 cells and found that the OCR of DOX-treated cells was significantly lower than that of the control (Figure 4C and Figure S1). These results suggest that CmP redox metabolism in DOX-treated myocardium was decreased because of mitochondrial dysfunction, at least in vitro.

## 4. Discussion

To the best of our knowledge, this is the first study to investigate the redox status of DOX-induced cardiomyopathy via in vivo DNP–MRI using the nitroxyl radical 3-carbamoyl-PROXYL as a molecular imaging probe. The reduction rate, as estimated from the enhanced DNP signal, was significantly slower in DOX-treated mice than in control mice 6 days post-DOX administration. To eliminate the effect of decreased cardiac output due to weight loss, we also determined the reduction rate of the DNP signal 2 days post-DOX-administration. The reduction rate of DOX-treated mice was significantly lower than that in control mice, even though there was no difference in the cardiac function according to the echocardiography results and in the rate of weight loss. These results suggest that the changes in redox metabolism detected by DNP–MRI occur earlier than those detected by physiological examination or clinical symptoms. Therefore, DNP–MRI, which can noninvasively detect mitochondrial oxidative stress and abnormalities, could be useful for early diagnosis of cardiomyopathy.

Since the heart is a luminal organ, it is possible that the DNP–MRI signal originates from the blood as well as the myocardium. Additionally, CmP is water-soluble and is excreted via urine, and changes in cardiac output and drug metabolism in renal plasma flow or glomerular filtration rate may alter its signal decay rate [40]. We measured the oxidized form and total CmP concentration (sum of reduced and oxidized forms) in the heart tissue and blood quantitatively using X-band EPR and confirmed that they did not change. Furthermore, even when smaller ROIs were used, the changes in the reduction rate in DNP–MRI were similar to those observed when ROIs of the entire heart were used in this study (data not shown). These results suggest that the reduction in the rate of reduction observed from the image intensity of DNP–MRI in DOX-treated mice is indicative of a change in the redox state of the myocardium. 

Next, we investigated the mechanism by which DOX injection in mice reduced the CmP reduction rate using cell models and DNP–MRI. The embryonic cardiomyocyte cell line H9c2 is commonly used in numerous in vitro studies, including cardiotoxicity analysis of drugs such as DOX [41,42]. The EPR signal change in H9c2 cells was inhibited by KCN, an inhibitor of complex IV in the mitochondrial ETC, as previously reported [27], suggesting that the redox metabolism of CmP in the myocardium is mitochondrion-dependent. We found that the EPR signal change in DOX-treated H9c2 cells was significantly suppressed compared to that in the control, suggesting that DOX suppressed the mitochondrial function in H9c2 cells. The myocardium has high mitochondrial content, and it has been reported that oxidative stress and abnormal mitochondrial function are observed in DOX-induced cardiomyopathy [8,9,10]. Previous studies have shown that the OCR is significantly suppressed in mitochondria isolated from DOX-treated hearts [43,44,45,46]. In this study, we found that the OCR of DOX-treated cells was significantly suppressed compared with that of the control. These results suggest that the changes in the redox metabolism of CmP in DOX-treated mice observed via DNP–MRI reflect mitochondrial dysfunction. A recent study has corroborated this inference by demonstrating that cytochrome c oxidase of mitochondrial complex IV is inhibited in non-alcoholic steatohepatitis [47], implying a relationship between the failure of mitochondrial ETC and the redox reaction of CmP [27]. This study has a limitation. The study aimed to develop early diagnostics for acute heart failure. Therefore, we used a mouse model of DOX-induced acute cardiomyopathy using a single dose of DOX. DOX causes long-term irreversible cardiomyopathy in clinical settings. In order to better understand irreversible chronic cardiomyopathy, it is necessary to examine, in future studies, chronic cardiomyopathy using a mouse model of DOX-induced cardiomyopathy with long-term multiple doses.

## 5. Conclusions

In this study, in vivo DNP–MRI using nitroxyl radicals was successfully performed to visualize the abnormal change in redox status in a DOX-induced cardiomyopathy mouse model. To date, most studies on oxidative stress and abnormal mitochondrial function have been conducted by measuring metabolites [48]; therefore, the specific time and location have not been precisely elucidated. We believe that our in vivo imaging indirectly compensates for this gap in knowledge by enabling the visualization of oxidative stress and mitochondrial dysfunction in real time. In recent years, cardiac MRI has evolved as a noninvasive test, and sequences such as T1 and T2 parametric maps can be used to characterize tissues for inflammation in clinical situations [49]. With the continued development of MRI, in vivo DNP–MRI could become a new and useful option for MRI diagnosis and monitoring of cardiomyopathy and myocarditis.

## Data Availability

The datasets used and analyzed in this study are made available through the corresponding author upon reasonable request.

## Supplementary Materials

The following supporting information can be downloaded at: https://www.mdpi.com/article/10.3390/antiox11081454/s1, Figure S1: Signal profiles of oxygen consumption in H9c2 cells obtained using the Mitochondrial Stress Test Complete Assay Kit.
