# Supplementary material for: Noninvasive Diagnosis of the Mitochondrial Function of Doxorubicin-Induced Cardiomyopathy Using In Vivo Dynamic Nuclear Polarization–Magnetic Resonance Imaging
Source: Antioxidants (Basel). 2022 Jul 26;11(8):1454. doi: 10.3390/antiox11081454 (PMC9331045; doi:10.3390/antiox11081454)
Supplement: Supplementary file 1 [file antioxidants-11-01454-s001.zip › antioxidants-1810914-supplementary.pdf]

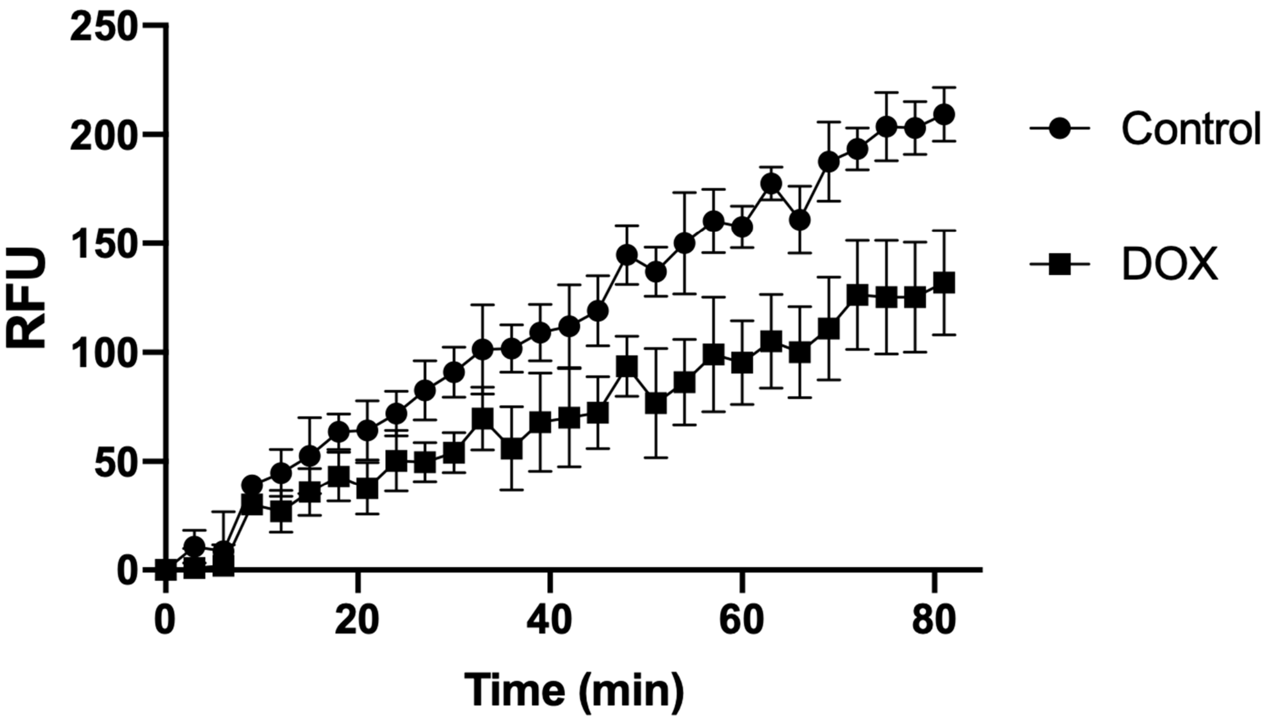

Figure S1. Signal profiles of oxygen consumption in H9c2 cells obtained using the Mitochondrial Stress Test Complete Assay Kit. The slopes derived from the linear part of the signal profile of the extracellular O_2 probe of cells treated with the kit components are indicative of oxygen consumption rates (n=3 in each group). DOX, doxorubicin; RFU, relative fluorescence unit.
